# Enhanced Reliability and Accuracy for Field Deployable Bioforensic Detection and Discrimination of *Xylella fastidiosa* subsp. *pauca*, Causal Agent of Citrus Variegated Chlorosis Using Razor Ex Technology and TaqMan Quantitative PCR

**DOI:** 10.1371/journal.pone.0081647

**Published:** 2013-11-29

**Authors:** Ping Ouyang, Mohammad Arif, Jacqueline Fletcher, Ulrich Melcher, Francisco Manuel Ochoa Corona

**Affiliations:** 1 National Institute for Microbial Forensics & Food and Agricultural Biosecurity, Oklahoma State University, Stillwater, Oklahoma, United States of America; 2 Department of Entomology and Plant Pathology, Oklahoma State University, Stillwater, Oklahoma, United States of America; 3 Department of Biochemistry and Molecular Biology, Oklahoma State University, Stillwater, Oklahoma, United States of America; Virginia Tech, United States of America

## Abstract

A reliable, accurate and rapid multigene-based assay combining real time quantitative PCR (qPCR) and a Razor Ex BioDetection System (Razor Ex) was validated for detection of *Xylella fastidiosa* subsp. *pauca* (*Xfp*, a xylem-limited bacterium that causes citrus variegated chlorosis [CVC]). CVC, which is exotic to the United States, has spread through South and Central America and could significantly impact U.S. citrus if it arrives. A method for early, accurate and sensitive detection of *Xfp* in plant tissues is needed by plant health officials for inspection of products from quarantined locations, and by extension specialists for detection, identification and management of disease outbreaks and reservoir hosts. Two sets of specific PCR primers and probes, targeting *Xfp* genes for fimbrillin and the periplasmic iron-binding protein were designed. A third pair of primers targeting the conserved cobalamin synthesis protein gene was designed to detect all possible *X. fastidiosa* (Xf) strains. All three primer sets detected as little as 1 fg of plasmid DNA carrying *X. fastidiosa* target sequences and genomic DNA of *Xfp* at as little as 1 - 10 fg. The use of Razor Ex facilitates a rapid (about 30 min) in-field assay capability for detection of all *Xf* strains, and for specific detection of *Xfp*. Combined use of three primer sets targeting different genes increased the assay accuracy and broadened the range of detection. To our knowledge, this is the first report of a field-deployable rapid and reliable bioforensic detection and discrimination method for a bacterial phytopathogen based on multigene targets.

## Introduction

*Xylella fastidiosa* (*Xf*), a xylem limited plant pathogen, causes a large number of diseases including plum leaf scald, phony peach, pear leaf scald, alfalfa dwarf, and leaf scorch of coffee, almond, elm, sycamore, oak, maple, mulberry, and oleander, but the two most economically important are Pierce’s disease of grapevines and citrus variegated chlorosis (CVC) [1,2]. Of four subspecies, only *X. fastidiosa* subsp. *pauca* (*Xfp*) does not occur in the United States [3]. *Xfp*, categorized as a select agent until 2012, causes CVC and coffee leaf scorch (CLS). In the early 1990s, the world’s largest citrus producer, Brazil, endured an outbreak of CVC that caused serious crop losses. After this outbreak, Brazilian researchers determined the complete genomic sequence of the CVC strain (9a5c) of *Xfp* [2,4], making it the first plant pathogenic bacterium to be completely sequenced [5,6]. According to Mansfield et al. [7] the pathogen ranked eighth among the 10 most important plant pathogenic bacteria, based on scientific and/or economic importance. Leaves of *Xfp-*infected citrus trees develop chlorotic spots on leaves and produce small, hard and juiceless fruits that lack commercial value, probably due to blockage of delivery of water and nutrients by aggregation of the bacteria as well as by the xanthan-like gum that the bacteria produce [8]. Citrus is produced in tropical and subtropical climates where the relatively high temperature and moisture are favorable for production. These same climatic conditions are also very advantageous for xylophagous sharpshooter leafhoppers and spittlebugs, which are important vectors of *Xfp* [9–15]. *Xfp* is considered a threat to the citrus industry in the U. S., and the U.S. Department of Agriculture (USDA) listed it as a quarantine select agent and considered it a high consequence pathogen. Timely diagnosis of CVC in the field is a challenge since it takes twelve months to develop the symptoms after the infection [16]. *In vitro* culture of all strains of *X. fastidiosa* is labor intensive and time consuming [17]. Thus, rapid discrimination of *Xfp* from other *X. fastidiosa* strains is essential for protecting the citrus industry. 

As an exotic microorganism with a high risk profile, we chose *Xfp* for the development of an enhanced detection method. Whether this pathogen were to be introduced naturally (weather, insect vector, birds etc.), unintentionally (trade, travel, etc.), or intentionally, rapid pathogen detection and disease diagnostic assays will be critical during the initial outbreak delimitation, as well as during follow-on implementation of management activities, when decision making will require specific, accurate and rapid identification of the pathogen. 

PCR based techniques are generally more sensitive than immunological methods and have high specificity and powerful discriminatory capabilities. Real-time qPCR offers greater sensitivity and speed compared to endpoint PCR for the detection of target DNA [18,19]. In field settings, however, plant pathogen detection can be challenging, since thermocyclers have limited sample capacity and require electrical power. The use of a portable, battery-operated real-time qPCR platform for in-field molecular testing allows minimally trained operators to test plant and environmental samples in the absence of laboratory facilities and conditions normally required, including electricity, centrifuges, liquid nitrogen, water baths, incubators and hazardous chemicals. Several portable instruments developed for this purpose include the Smart Cycler (Cepheid, Sunnyvale, CA), the LightCycler (Roche Applied Science, Indianapolis, IN), the Razor Ex Biodetection System (Razor Ex; Idaho Technology Inc.), and the Bio-Seeq instrument (Smiths Detection, Edgewood, MD). 

In 2002, Schaad et al. used the Smart Cycler system to detect *X. fastidiosa* in sap from asymptomatic grapevines in two hours [20]. The Smart Cycler also has been applied in the identification of *Phytophthora ramorum*, which causes sudden oak death [21], and the *Aphthovirus* that causes foot-and-mouth disease [22]. The Razor Ex was designed originally to allow first responders and front line military operations to identify biological threat organisms on-site. The Razor Ex system offers ready-to-use, freeze-dried reagent pouches, barcode-based PCR cycling program upload and Bluetooth capabilities for wireless data transmission. Due to fast cycling parameters, Razor Ex takes about only 30-40 minutes compared to a traditional PCR using the ABI 7300/7500 thermocycler (Applied Biosystems, Foster City, CA) that takes about 100 minutes. A Razor Ex based method also detected influenza A viruses near the patient’s location and with sensitivity and specificity similar to those of the ABI 7300 [23–25]. 

Developing an assay for a select agent presents further challenges. We here report the development of such an assay for a pathogen that was on the select agent list during the course of development of the assay and should thus serve as a model for developing such future field detection procedures for regulated organisms. Specifically, field deployable, rapid TaqMan qPCR and Razor Ex protocols for reliable, sensitive, and accurate detection of *X. fastidiosa* and *Xfp* based on three discriminatory genome segments. This detection system will enhance investigative capability for ecological, agriculture and/ or biosecurity and microbial forensics. 

## Materials and Methods

### Ethics statement

All samples included in the exclusivity and inclusivity panels of this research were used with permission from concerned persons, scientists and diagnosticians who provided these samples. This research did not involve endangered or protected species.

### Sources of inclusivity and exclusivity panels

Infected plants from which genomic DNA was extracted for use in the inclusivity and/or exclusivity panels are shown in Table 1. Microbes included in the exclusivity panel are presented in Table 2. Members of the plant exclusivity panel, including *Medicago sativa, Arabidopsis thaliana, Hordeum vulgare, Zea mays, Gossypium hirsutum, Lens culinaris, Avena sativa, Petroselinum crispum, Arachis hypogaea, Solanum tuberosum, Secale cereale, Sorghum bicolor, Glycine max, Helianthus annuus, Nicotiana tabacum, Lycopersicon esculentum* and *Triticum aestivum*, were grown in a BSL-2 greenhouse at the Noble Research Center, Oklahoma State University, Stillwater, OK. Other members of the plant exclusivity panel, including *Vitis aestivalis, Prunus persica, Carya illinoinensis*, were obtained from the Cimarron Valley Research Station, Perkins, OK; *Nephrolepis exaltata* was collected from an indoor garden at Oklahoma State University; *Citrus sinensis* and *Rosa* species were obtained from F. M. Ochoa-Corona, Oklahoma State University. Foliar tissues of each plant species were used for DNA isolation except *C. sinensis* from which rind was used. Jiffy soil mix (Ferry Morse Seed Co., Fulton, KY) was also included in exclusivity panel.

**Table 1:** DNA sources used for validation of *Xylella fastidiosa* and *Xylella fastidiosa* subsp. *pauca* specific primer and probe sets.

Sample code	Acronym of target pathogen	Host plant/insect	Origin	Source	Ct values with specific primer and probe sets		
					Xf.CVC.fim1_(SD)_	Xf.CVC.pib4_(SD)_	Xf.csp6_(SD)_
Xf_k	*Xf*	Grape	Texas, USA	B. Bextine, UT Tyler, TX	-	-	22.9_(0.12)_
Xf_g	*Xf*	Grape	Texas, USA	B. Bextine, UT Tyler, TX	-	-	30.4_(0.25)_
TX PD1	*Xf*	Grape	Texas, USA	B. Bextine, UT Tyler, TX	-	-	25.06_(0.26)_
TX PD2	*Xf*	Grape	Texas, USA	B. Bextine, UT Tyler, TX	-	-	21.22_(0.06)_
F12	*Xff*	Sharpshooter	Texas, USA	B. Bextine, UT Tyler, TX	-	-	32.29_(0.28)_
F15	*Xff*	Sharpshooter	Texas, USA	B. Bextine, UT Tyler, TX	-	-	28.56_(0.24)_
M1	*Xfm*	Sharpshooter	Texas, USA	B. Bextine, UT Tyler, TX	-	-	36.64_(0.68)_
M2	*Xfm*	Sharpshooter	Texas, USA	B. Bextine, UT Tyler, TX	-	-	35.13_(1.08)_
200901617	*Xf*	Grape	Oklahoma, USA	J. Olsen, PDIDL, OSU, OK	-	-	22.5_(0.02)_
20092259	*Xf*	Grape	Oklahoma, USA	J. Olsen, PDIDL, OSU, OK	-	-	29.7_(0.06)_
200901779	*Xf*	Grape	Oklahoma, USA	J. Olsen, PDIDL, OSU, OK	-	-	21.0_(0.06)_
200902412	*Xf*	Grape	Oklahoma, USA	J. Olsen, PDIDL, OSU, OK	-	-	22.50_(0.07)_
200902348	*Xf*	Grape	Oklahoma, USA	J. Olsen, PDIDL, OSU, OK	-	-	30.97_(0.31)_
201101794	*Xf*	Grape	Oklahoma, USA	J. Olsen, PDIDL, OSU, OK	-	-	29.0_(0.26)_
200901990	*Xf*	Grape	Oklahoma, USA	J. Olsen, PDIDL, OSU, OK	-	-	33.72_(0.33)_
C178	*xf*	Grape	Oklahoma, USA	J. Olsen, PDIDL, OSU, OK	-	-	19.6_(0.17)_
200901994	*Xf*	Grape	Oklahoma, USA	J. Olsen, PDIDL, OSU, OK	-	-	21.4_(0.04)_
200902414	*Xf*	Grape	Oklahoma, USA	J. Olsen, PDIDL, OSU, OK	-	-	27.2_(0.07)_
C121D	*Xf*	Oak	Oklahoma, USA	J. Olsen, PDIDL, OSU, OK	-	-	17.0_(0.16)_
C85D	*Xf*	Oak	Oklahoma, USA	J. Olsen, PDIDL, OSU, OK	-	-	18.97_(0.19)_
C177D	*Xf*	Elm	Oklahoma, USA	J. Olsen, PDIDL, OSU, OK	-	-	25.1_(0.11)_
C88D	*Xf*	Elm	Oklahoma, USA	J. Olsen, PDIDL, OSU, OK	-	-	28.94_(0.34)_
C173D	*Xf*	Mulberry	Oklahoma, USA	J. Olsen, PDIDL, OSU, OK	-	-	21.52_(0.18)_
C83D	*Xf*	Mulberry	Oklahoma, USA	J. Olsen, PDIDL, OSU, OK	-	-	19.24_(0.17)_
Temecula*	*Xft*	Grape	California, USA	ATCC, Manassas, VA	-	-	13.6_(0.31)_
CVC50024*	*Xfp*	Citrus	Brazil	D. Luster, USDA-ARS, FDWSRU, Fort Detrick, MD	18.8_(0)_	20.2_(0.03)_	16.6_(0.02)_
CVC50031*	*Xfp*	Citrus	Brazil	D. Luster, USDA-ARS, FDWSRU, Fort Detrick, MD	18.5_(0.06)_	22.2_(0.1)_	18.6_(0.07)_

* Genomic DNA from pure culture; FDWSRU = Foreign Diseases and Weeds Research Unit; PDIDL = Plant Disease Diagnostic Laboratory; ATCC = American Type Culture Collection; OSU = Oklahoma State University; SD = replicates Ct standard deviation; - = no amplification (negative); *Xf* = *Xylella fastidiosa; Xfp* = *Xylella fastidiosa* subsp. *pauca; Xft = Xylella fastidiosa* Temecula*; Xfm* = *Xylella fastidiosa* subsp. *multiplex; Xff* = *Xylella fastidiosa* subsp. *fastidiosa.*

**Table 2:** Members of microbial panel used for validation of primer and probe sets Xf.CVC.fim1, Xf.CVC.pib4 and Xf.Csp6 specific for *Xylella fastidiosa* subsp. *pauca* and *Xylella fastidiosa* in general.

Microbes	Specific primers and probes			Source
	Xf.CVC.fim1	Xf.CVC.pib4	Xf.Csp6	
*Burkholderia cepacia* (ATCC 25416)	-	-	-	ATCC, Manassas, VA
*Pseudomonas syringae* *pv. syringae* (ATCC 33291)	-	-	-	ATCC, Manassas, VA
*Escherichia coli* (1472)	-	-	-	S. Gilliland, OSU, Stillwater, OK
*Pseudomonas fluorescens* (ATCC 13525)	-	-	-	ATCC, Manassas, VA
*Pseudomonas syringae* *pv. phaseolicola* (1448a)	-	-	-	C. Bender, OSU, Stillwater, OK
*Phytophthora capsici*	-	-	-	S. M. Marek, OSU, Stillwater, OK
*Xanthomonas vesicatoria* (ATCC 35937)	-	-	-	ATCC, Manassas, VA
*Ralstonia solanacearum* (ATCC 11696)	-	-	-	ATCC, Manassas, VA
*Erwinia tracheiphila*	-	-	-	B. Bruton, USDA-ARS, Lane, OK
*Xanthomonas citri* subsp*. Citri* (Xcc A 306)	-	-	-	N. Jalan, UF, Lake Alfred, FL
*Xanthomonas citri* subsp*. Citri* (Xcc Aw 12879)	-	-	-	N. Jalan, UF, Lake Alfred, FL
*Xanthomonas citri* subsp. *Citri* (Xcc A 270)	-	-	-	N. Jalan, UF, Lake Alfred, FL
*Xanthomonas axonopodis* pv.citrumela (acm FL1)	-	-	-	N. Jalan, UF, Lake Alfred, FL
Phymatotrichopsis omnivora*	-	-	-	F. M. Ochoa-Corona, OSU, Stillwater, OK
*Pythium aphanidermatum*	-	-	-	C. Garzon, OSU, Stillwater, OK
Non-template control (water)	-	-	-	Ambion, Austin, TX
Positive control**	+	+	+	Generated in NIMFFAB laboratory through TOPO-TA cloning of target sequence

- = No amplification (negative); + = amplification (positive); *DNA from alfalfa infected with *P. omnivora*; **plasmid DNA carrying the target gene sequence; ATCC = American Type Culture Collection; OSU = Oklahoma State University; UF = University of Florida

### DNA isolation from plants and microbes

Genomic DNAs of *X. fastidiosa* and infected plant/insect samples were obtained from the American Type Culture Collection (Manassas, VA) and from governmental and university laboratories (Table 1). Genomic DNA from plants was extracted using a DNeasy Plant Mini Kit (Qiagen, Valencia, CA) and bacterial DNA (Table 2) was extracted using the Qiagen DNeasy Blood & Tissue Kit (Qiagen) following the manufacturer’s instructions. Crude DNA from sharpshooters (*Homalodisca vitripennis*) was isolated using prepGEM™ (ZyGEM Corporation Ltd, Hamilton, New Zealand) following the manufacturer’s protocol. The concentrations of total genomic DNAs were determined using a NanoDrop v.2000 spectrophotometer (Thermo Fisher Scientific Inc., Worcester, MA). For field application, isolation of *X. fastidiosa* DNA from infected plant samples (grape and oak; two samples each) to be tested with the Razor Ex was done using the Dynabeads-based modified method developed for the fungus *P. omnivora* [25] using Dynabeads DNA Direct Universal Kit (Invitrogen, Carlsbad, CA). Briefly, 10 to 30 mg infected foliar tissues were macerated in 100 to 150 µl Tris-EDTA (TE) buffer (Promega, Madison, WI). The mixture of 40 µl of macerated supernatant and 200 µl of Dynabeads was incubated for 5 min at RT. Tubes containing this mix were placed in a magnetic rack to retain the beads while the liquid was discarded. The beads were rinsed twice with wash buffer. Manufacturer provided suspension buffer was added to suspend DNA. 

### Primer and probe design

The genes for fimbrillin, periplasmic iron-binding protein, and cobalamin were targeted. The first two were used for specific detection of *Xfp* and the third for specific detection of all strains of *X. fastidiosa*. Three optimal primer and probe sets were designed following the parameters described by Arif and Ochoa-Corona [26] as shown in Table 3. The complete genome of *Xfp* 9a5c (accession number AE003849), retrieved from the NCBI GenBank database (http://www.ncbi.nlm.nih.gov/), was used to subtract the *Xfp* specific sequences using MUMmer software [27]. Two primer and probe sets, Xf.CVC.fim and Xf.CVC.pib, specific to *Xfp* were designed using Primer3 [28]. The cobalamin synthesis protein gene sequence (accession number CP002165) was retrieved from the GenBank database and a primer set, Xf.csp6, was designed from it to detect all strains of *X. fastidiosa*. The primer pair Xf.csp.6F/Xf.csp.6R and the Xf.csp.6 probe were aligned with whole genome sequences of *X. fastidiosa* subsp. *fastidiosa* GB514 (accession number CP002165), *X. fastidiosa* M23 (accession number CP001011), *X. fastidiosa* M12 (accession number CP000941), *X. fastidiosa* Temecula1 (accession number AE009442), and *Xfp* 9a5c (accession number AE0003849), all available in GenBank. Primer thermodynamics, internal structures, and self-dimer formation were examined *in silico* with mFold [29]. The specificity was confirmed *in silico* by screening the primer and probe sequences with BLASTn, available from the GenBank nucleotide database [30] (Table 3). Primers and double quencher probes-linked 5’ 6-carboxyfluorescein/ZEN^TM^/3’ Iowa Black FQ (5’ 6-FAM^TM^/ZEN^TM^/3’ IB^®^FQ) were synthesized by IDT (Integrated DNA Technologies, Inc., Coralville, IA). 

**Table 3:** Specific primers and probes used for PCR amplification of *Xylella fastidiosa* and *Xylella fastidiosa* subsp. *pauca*.

Primer and probe name	Acronym of target pathogen	Primer/probe sequence (5’-3’)	Targeted gene	Amplicon size (bp)	G + C%	*ΔG	NCBI BLASTn E-value
Xf.CVC.fim1F	*Xfp*	TGACCTGGATATGTATTACGAACCT	Fimbrillin	109	40	0.9	2e-04
Xf.CVC.fim1R		TAGACGCACGGTGGTTTTGT			50	1.0	0.067
Xf.CVC.fim1P		TGGTGTTTGAGGGAGGGCATCTGT			54	0.9	7e-04
Xf.CVC.pib4F	*Xfp*	CATTCAAGGTTCCAACGACTT	Periplasmic iron-binding protein	81	43	0.9	0.025
Xf.CVC.pib4R		GGTCACTTTAGTTCCAGGATGC			50	0.2	0.006
Xf.CVC.pib4P		CATGTTTGCTTTGGTGATTGCTGATT			38	0.9	6e-05
Xf.Csp6F	*Xf*	CCCATTACGCTTCAACCATT	Cobalamin synthesis protein	93	45	0.2	0.067
Xf.Csp6R		CCCAATCCATACGACTTGCT			50	0.6	0.067
Xf.Csp6P		GGTGTGATTCGCAGCAAGGGC			62	0.8	0.025

* ΔG value from plot calculated by mFOLD at 60°C; *Xf* = *Xylella fastidiosa; Xfp* = *Xylella fastidiosa* subsp. *pauca*

### PCR and qPCR amplification

Preliminary PCR assays were carried out in an Eppendorf thermal cycler (Eppendorf, Hauppauge, NY) using 20 μl reaction mixtures containing 10 μl GoTaq Green Master Mix (Promega), 1 μl of each forward and reverse primer from working stock of 5 µM, 1 μl of template DNA, and 7 μl nuclease free water. The cycling parameters consisted of 35 cycles as follows: Initial denaturation for 3 min at 94 °C followed by denaturation at 94 °C for 20 s, annealing at 56 °C for 30 s, extension 72 °C for 30 s, and final extension at 72 °C for 3 min. Plasmid DNA containing the target fragment and nuclease free water (non-template) were used as positive and negative controls, respectively, in each PCR amplification. Amplified PCR products were electrophoresed in a 1.5 % agarose gel in 1X TAE buffer, and amplicon sizes were estimated using 1kb plus ladders (Invitrogen). 

The qPCR assays were performed in a Rotor-Gene 6000 thermocycler (Corbett Research, Sydney, Australia) and the results were analyzed using the Rotor-Gene 6000 series software 1.7 (Built 87). qPCR assays were carried out in 20 μl reaction mixtures containing 10 μl of Platinum qPCR SuperMix-UDG (Invitrogen), 0.8 μl from working stock of 5 µM of each forward and reverse primer, 0.8 μl probe from working stock of 5 µM, 0.3 mg per ml (0.12 μl) BSA (Invitrogen), 1 μl of template DNA, and 6.48 μl of nuclease free water. Rotor-Gene qPCR cycling conditions were: two initial holds, each for 2 min at 50 °C and 95 °C, followed by 40 cycles at 95 °C for 15 s and 60 °C for 1 min. A minimum of three dilutions of plasmid DNA (positive control; carrying the target gene sequence) were used to generate a standard curve and negative (non-template; water) controls were included in each round of qPCR amplification. Each reaction with each member of inclusivity and exclusivity panel was performed in three replicates (same reaction mixture in three tubes) using each primer and probe set. 

### Razor Ex amplification

**Figure 1:**
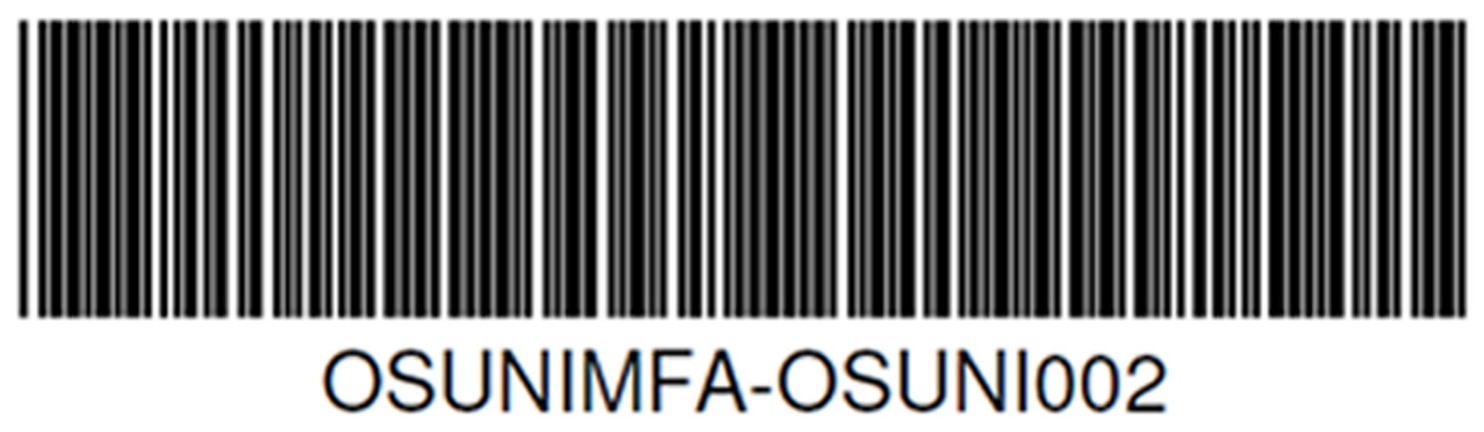
Bar code generated to upload the fast PCR cycling program for detection of *Xylella fastidiosa* and *Xylella fastidiosa* subsp. *pauca* in Razor Ex BioDetection system.

Amplification with each primer set was carried out in 150 μl reaction mixtures containing 75 μl of Platinum® Quantitative PCR SuperMix-UDG, 6.0 μl of each forward (biotinylated) and reverse primer from working stock of 5 µM, 6.0 μl probe from working stock of 5 µM, 4 μl (infected plant DNA) or 1 μl (pathogen genomic DNA) of template and nuclease free water to make up the volume. Positive (plasmid DNA; carrying the target gene sequence) and negative (non-template; water) controls were included in each Razor Ex amplification. Short cycling parameters included one initial hold for 2 min at 50°C, a first cycle at 94°C for 4 min and 60°C for 15 sec followed by 54 cycles at 91°C for 3 sec and 60°C for 15 sec. The PCR cycling program was uploaded using a barcode (Figure 1). The assays were performed in a Razor Ex BioDetection System.

### Real time qPCR sensitivity assays

The detection limits of all three primer and probe sets was determine by performing four sensitivity assays with each set of primer and probe in the Rotor-Gene 6000 thermocycler. Ten-fold serial dilution of plasmid or genomic DNA (*Xfp*) was used at 10 ng to 1 fg per reaction. For each spiked or mixed assay, 1 µl (per reaction) of rind extract (1 ml of TE buffer was used to macerate healthy orange rind and clarified by a 2 min centrifugation at 14,000 rpm; the supernatant was used) was added into serially diluted *Xfp* genomic DNA. A sensitivity assay spiked with sharpshooter DNA was performed by adding 1 µl (10 ng/µl) crude DNA of sharpshooter into serially diluted *Xfp* genomic DNA. Each reaction was performed in three replicates.

### Positive controls

Positive controls carrying target gene segments of *Xfp* and *Xf* were generated for each primer set targeting three different genes. The amplicons generated using endpoint PCR were eluted from the agarose gel using Quantum Prep Freeze 'N Squeeze Spin Columns (Bio-Rad, Hercules, CA) and inserted into a plasmid pCR2.1-TOPO vector (TOPO-TA Cloning kit; Invitrogen). QIAprep Spin Miniprep Kit (Qiagen) was used to purify plasmid DNA carrying the target sequence from overnight bacterial cultures for each primer set. Specific PCR amplicons for each target gene from isolated plasmid DNAs were sequenced by the Oklahoma State University Recombinant DNA/Protein Resource Facility using M13F and M13R primers. BLASTn tool of NCBI was used to check amplicons sequences against the GenBank nucleotide database. 

## Results

### Primer and probe design

The two primer sets specific for *Xfp* and the primer set for general detection of *X. fastidiosa* as well as all respective probes met the desired 100% query coverage and 100% identity after an alignment using BLASTn in the GenBank nucleotide database (Table 3). All primers and probes had ΔG ≤ 1.0 at 60°C (Table 3). To maximize signal and minimize background, the double-quenched probes contained a 5’ FAM fluorophore, a 3’ IBFQ quencher, and an internal ZEN quencher. 

### Real time qPCR protocol validation

All three primer and probe sets (Table 3) were designed to perform in endpoint PCR, qPCR, and the field-deployable Razor Ex. Primer and probe specificity was tested against a plant exclusivity panel (described above) and near-neighbor microbial panel (Table 2), and broad range detection of primer/probe set Xf.csp6 was tested against an inclusivity panel (Table 1) of *X. fastidiosa* genomic DNA from purified *Xf* isolates and infected plants and sharpshooters. Genomic DNA of only two *Xfp* isolates was available, for use in the inclusivity panel for primer and probe sets, Xf.CVC.fim1 and Xf.CVC.pib4, because *Xfp* was classified as a select agent at the time of these experiments. The primer and probe sets Xf.CVC.fim1, Xf.CVC.pib4 and Xf.csp6 showed no cross reactivity with any member of the exclusivity panel, and generated the expected 109, 81 and 93 bp PCR amplicons, respectively. The primer/probe sets Xf.CVC.fim1, and Xf.CVC.pib4 amplified only from *Xfp* while set Xf.csp6 amplified all *X. fastidiosa* (Table 1). To further confirm the specificity, the amplified products were cloned, sequenced, and assessed using BLASTn against the GenBank database. All the sequences showed highest similarity with the corresponding pathogen. Twenty symptomatic plants, three genomic DNAs from *Xfp* and *X. fastidiosa* Temecula, and four sharpshooters infected with *X. fastidiosa* subsp. *multiplex* and *X. fastidiosa* subsp. *fastidiosa* tested positive with primer/probe set Xf.csp6, while only *Xfp* isolates were positive using primer/probe sets Xf.CVC.fim1 and Xf.CVC.pib4 (Table 1). 

### qPCR sensitivity and spiked assays

**Figure 2:**
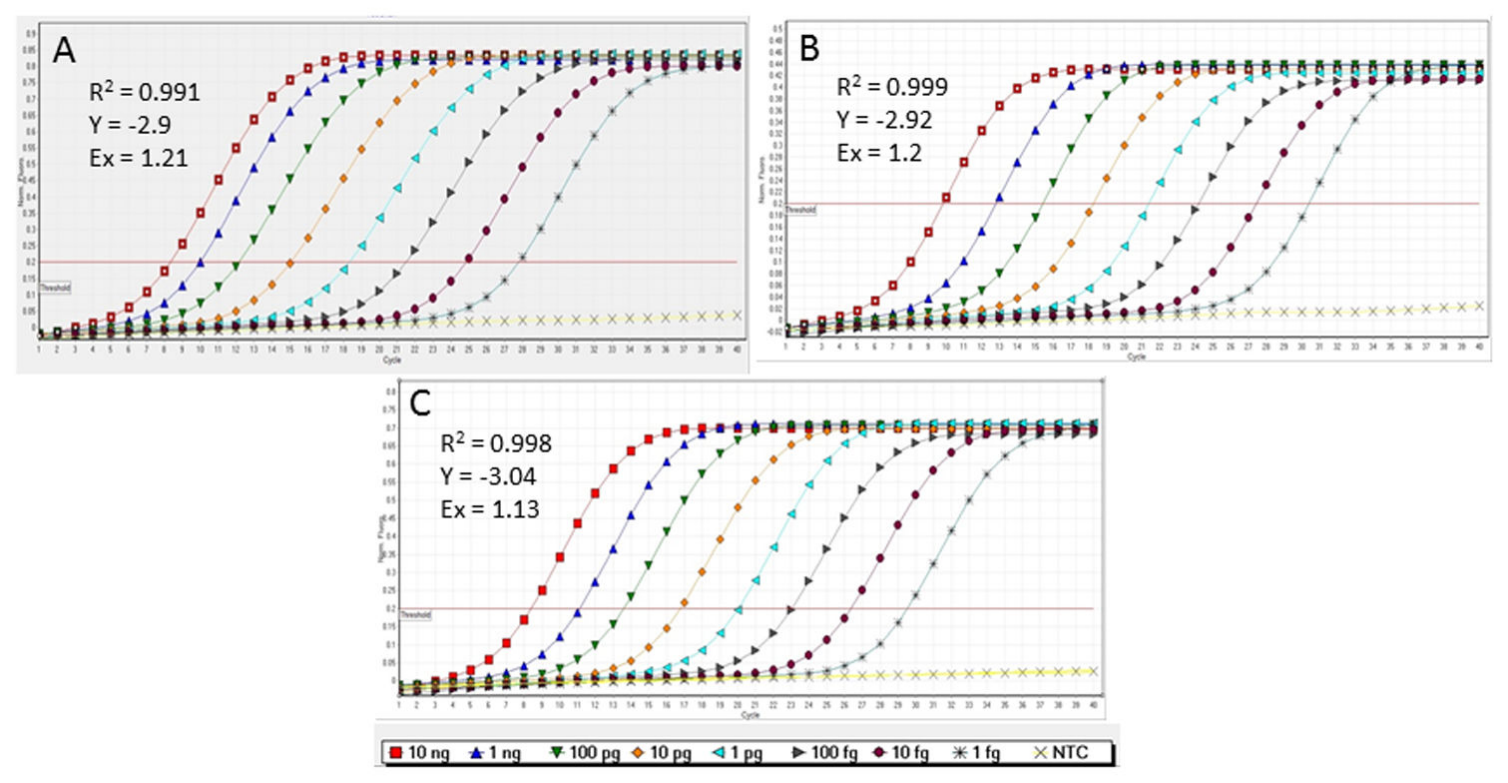
Standard graph showing 10-fold serial dilutions of plasmid DNA (carrying the target gene sequence of corresponding primer set) using primer and probe sets. Xf.CVC.fim1 (A), Xf.CVC.pib4 (B), and Xf.csp6 (C). R2 = linear correlation; Ex = reaction efficiency; Y = slope.

**Figure 3:**
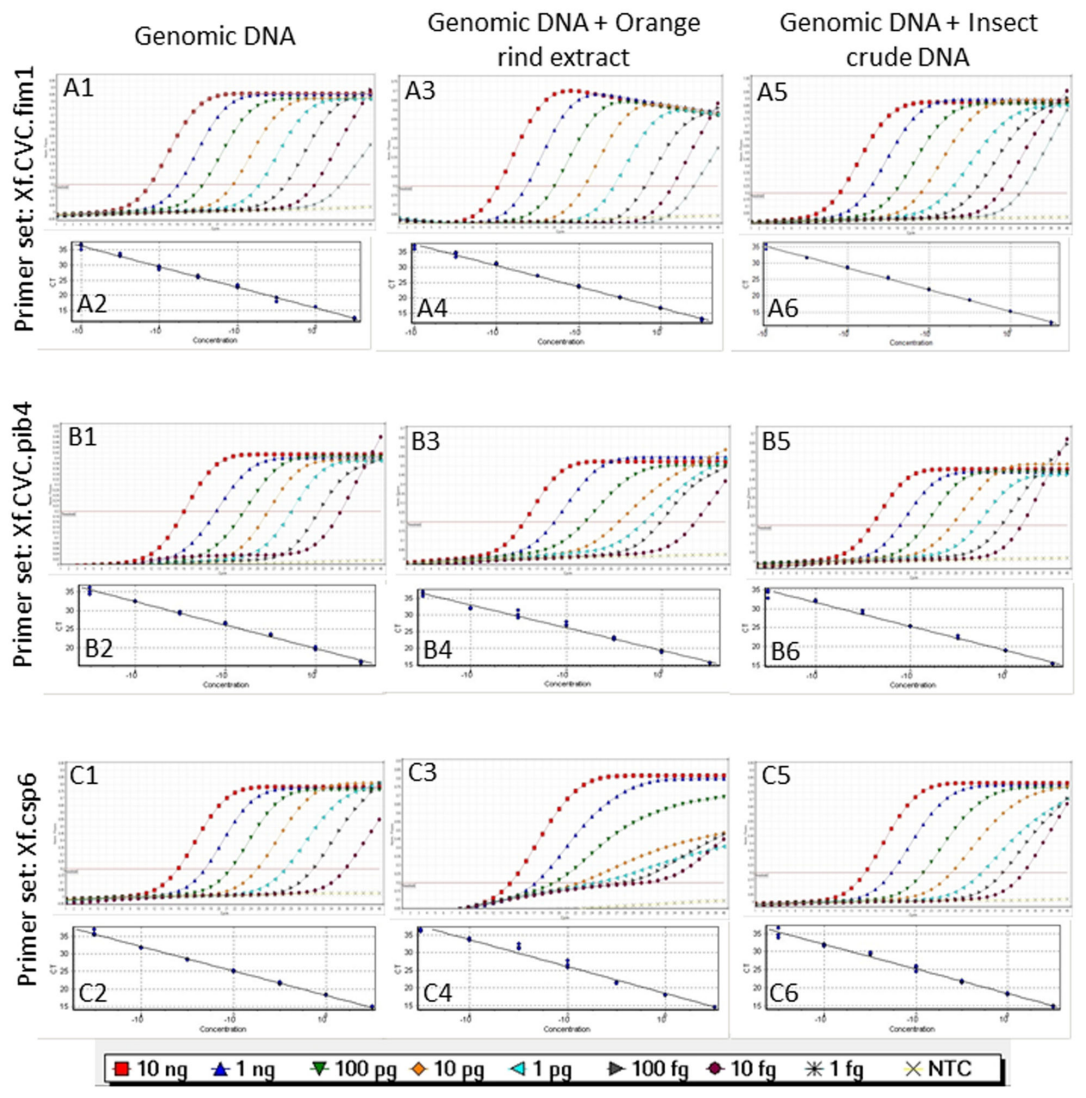
Standard curves and graphs generated using 10-fold diluted genomic DNA and genomic DNA mixed with orange rind extract or insect crude DNA. A1/A2, A3/A4 & A5/A6: Graphs/standard curve generated using primer and probe set Xf.CVC.fim1 with genomic DNA, genomic DNA mixed with orange rind extract and genomic DNA mixed with insect crude DNA, respectively; B1/B2, B3/B4 & B5/B6: Graphs/standard curve generated using primer/ probe set Xf.CVC.pib4 with genomic DNA, genomic DNA mixed with orange rind extract and genomic DNA mixed with insect crude DNA, respectively; C1/C2, C3/C4 & C5/C6: Graphs/standard curve generated using primer/ probe set Xf.csp6 with genomic DNA, genomic DNA mixed with orange rind extract and genomic DNA mixed with insect crude DNA, respectively.

Primer sets Xf.CVC.fim1, Xf.CVC.pib4, and Xf.csp6 detected as little as 1 fg of plasmid DNA carrying *X. fastidiosa* target sequences at cycle threshold (Ct) values of 27.92, 30.19 and 29.55, respectively (Table 4; Figure 2). Almost identical Ct values, ranging from 8.37 to 9.73, were obtained from 10 ng of plasmid DNA. The obtained linear graphs and standard curve values for each primer-probe set used to amplify corresponding positive control, suggest optimal reaction efficiency (Table 4; Figure 2). The detection limit of primer-probe set Xf.CVC.fim1 reached as little as 1 fg; (Table 4; Figure 3) compared to those of primer and probe sets Xf.CVC.pib4 and Xf.csp6 that detected as little as 10 fg with *Xfp* genomic DNA (Table 4; Figure 3). A small difference in sensitivity and a variation among the replicates at lower concentration (especially at 10 fg and below) of genomic DNA was observed when a *Xfp* genomic DNA sample was mixed separately with extracts of orange rind and a crude sharpshooter DNA preparation (Table 4; Figure 3). Primer and probe set Xf.CVC.fim1 was able to detect down to 1 fg of genomic DNA of *Xfp* (CVC50031) mixed with extracts of orange rind and crude sharpshooter DNA but showed a variation in Ct values among the replicates (Figure 3). To comparing standard graphs generated for all the three primer and probe sets, a manual normalized fluorescence value of 0.2 was used. Generated standard graph values suggested that orange rind extract and crude sharpshooter DNA have little or no inhibitory effect on qPCR sensitivity when the spiked and non-spiked sensitivity assays were performed using all three primer/probe sets (Table 4). 

**Table 4:** Average Ct values of sensitivity and spiked assays using primer/probe sets Xf.CVC.fim1, Xf.CVC.pib4 and Xf.csp6.

	Template conc. per reaction	*Number of genomic DNA copies	Plasmid DNA			Genomic DNA			Genomic DNA spiked with rind extract			Genomic DNA spiked with crude sharpshooter DNA		
			Xf.CVC.	Xf.CVC.	Xf.csp6	Xf.CVC.	Xf.CVC.	Xf.csp6	Xf.CVC.	Xf.CVC.	Xf.csp6	Xf.CVC.	Xf.CVC.	Xf.csp6
			fim1	pib4		fim1	pib4		fim1	pib4		fim1	pib4	
**R^2^**	-	-	0.991	0.999	0.998	0.996	0.994	0.997	0.996	0.985	0.980	0.998	0.991	0.991
**Y**	-	-	-2.9	-2.92	-3.04	-3.39	-3.17	-3.49	-3.48	-3.40	-3.8	-3.31	-3.14	-3.39
**Ex**	-	-	1.21	1.2	1.13	0.97	1.07	0.94	0.94	0.97	0.83	1.01	1.08	0.97
**Ct_(SD)_ values^**	10 ng	3.46x10^6^	8.37_(0.06)_	9.73_(0.08)_	8.41_(0.02)_	12.43_(0.23)_	16.03_(0.27)_	14.84_(0.08)_	12.91_(0.36)_	15.51_(0.13)_	14.52_(0.12)_	11.79_(0.28)_	15.49_(0.10)_	14.85_(0.14)_
	1 ng	3.46x10^5^	9.98_(0.02)_	12.83_(0.02)_	11.16_(0.07)_	16.06_(0.04)_	19.82_(0.35)_	18.15_(0.12)_	16.53_(0.09)_	18.98_(0.27)_	17.98_(0.12)_	15.12_(0.09)_	18.94_(0.09)_	18.29_(0.25)_
	100 pg	3.46x10^4^	12.17_(0.02)_	15.35_(0.06)_	13.56_(0.08)_	18.78_(0.69)_	23.39_(0.18)_	21.56_(0.35)_	20.06_(0.04)_	22.84_(0.37)_	21.40_(0.17)_	18.67_(0.10)_	22.4_(0.37)_	21.71_(0.30)_
	10 pg	3.46x10^3^	15.07_(0.03)_	18.22_(0.04)_	16.77_(0.06)_	22.95_(0.42)_	26.43_(0.21)_	25.07_(0.21)_	23.68_(0.26)_	27.2_(0.66)_	26.62_(0.93)_	21.88_(0.12)_	25.38_(0.17)_	25.30_(0.81)_
	1 pg	3.46x10^2^	18.48_(0.15)_	21.32_(0.15)_	20.02_(0.03)_	26.16_(0.32)_	29.23_(0.35)_	28.41_(0.10)_	27.24_(0.01)_	30.16_(1.20)_	31.67_(0.71)_	25.49_(0.28)_	29.17_(0.38)_	29.37_(0.25)_
	100 fg	3.46x10^1^	21.55_(0.03)_	24.19_(0.09)_	22.92_(0.13)_	29.02_(0.53)_	32.43_(0.16)_	31.69_(0.11)_	31.13_(0.25)_	31.84_(0.28)_	33.57_(0.25)_	28.54_(0.21)_	31.90_(0.16)_	31.77_(0.29)_
	10 fg	3.46x10°	25.00_(0.14)_	27.39_(0.04)_	26.42_(0.09)_	33.19_(0.43)_	35.22_(0.88)_	36.06_(0.78)_	34.21_(0.79)_	36.24_(0.71)_	36.17_(0.29)_	31.59_(013)_	33.92_(1.07)_	34.97_(1.37)_
	1 fg	3.46x10^-1^	27.92_(0.11)_	30.20_(0.33)_	29.55_(0.14)_	36.53_(0.90)_	NA	NA	36.47_(0.81)_	NA	NA	34.89_(1.02)$_	NA	NA
	NTC	-	NA	NA	NA	NA	NA	NA	NA	NA	NA	NA	NA	NA

*Number of copies were calculated according to the 2.679 Mb genome size of *Xylella fastidiosa* subsp. *pauca* (9a5c; GenBank accession number AE003849) using online calculator (http://cels.uri.edu/gsc/resources/cndna.html); ^average Ct (threshold cycle) value of three replicates; **^*$*^**mean of only two replicates; SD = standard deviation; NA = no amplification, R^2^= linear correlation; Ct = cycle threshold; Ex = reaction efficiency; Y = slop; NTC = non template control (water). Plasmid DNA concentration includes the total mass of plasmid DNA containing the target gene sequence of the corresponding primer/probe set. Plant-spiked assays with rind extract contain 1 µl of rind extract per reaction *H. vitripennis*-spiked assays contain 10 ng crude sharpshooter DNA per reaction.

### Razor Ex BioDetection System

**Figure 4:**
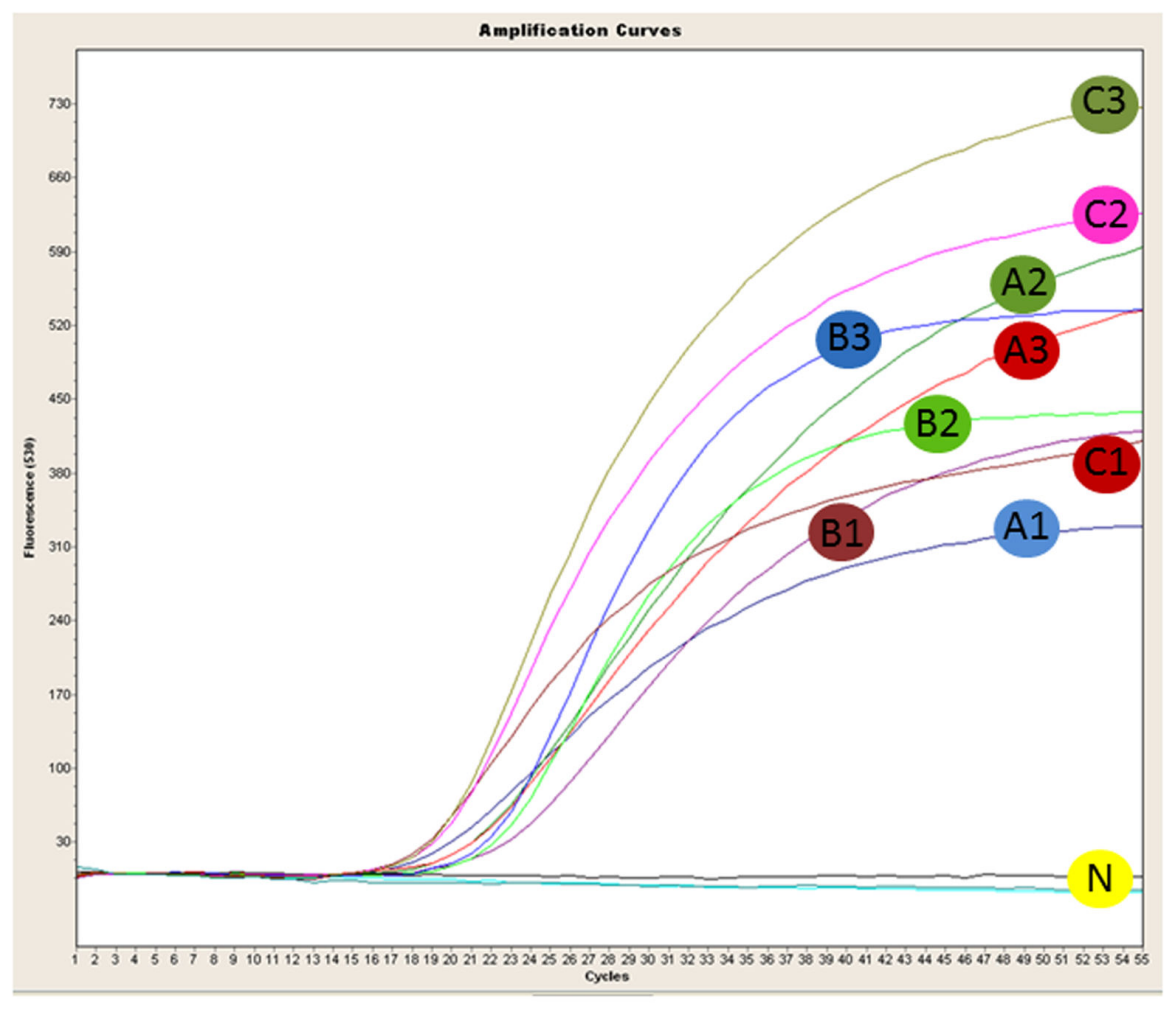
Razor Ex BioDetection system graph obtained after amplification of plasmid DNA (carrying the target gene sequence of corresponding primer set) and *Xylella fastidiosa* subsp. *pauca* genomic DNA (CVC50031) using primer and probe sets Xf.CVC.fim1, Xf.CVC.pib4 and Xf.csp6. A1, B1 and C1 are positive controls with estimated Ct values of 23, 26 and 20 for primer/probe sets Xf.CVC.fim1, Xf.CVC.pib4 and Xf.csp6, respectively. *X. fastidiosa* genomic DNA tested in two replicates with primer/probe sets Xf.CVC.fim1 (A2 & A3), Xf.CVC.pib4 (B2 & B3) and Xf.csp6 (C2 & C3) with estimated Ct value of 24, 24 and 20, respectively. N shows the non-template controls (water) for each primer/probe set.

**Figure 5:**
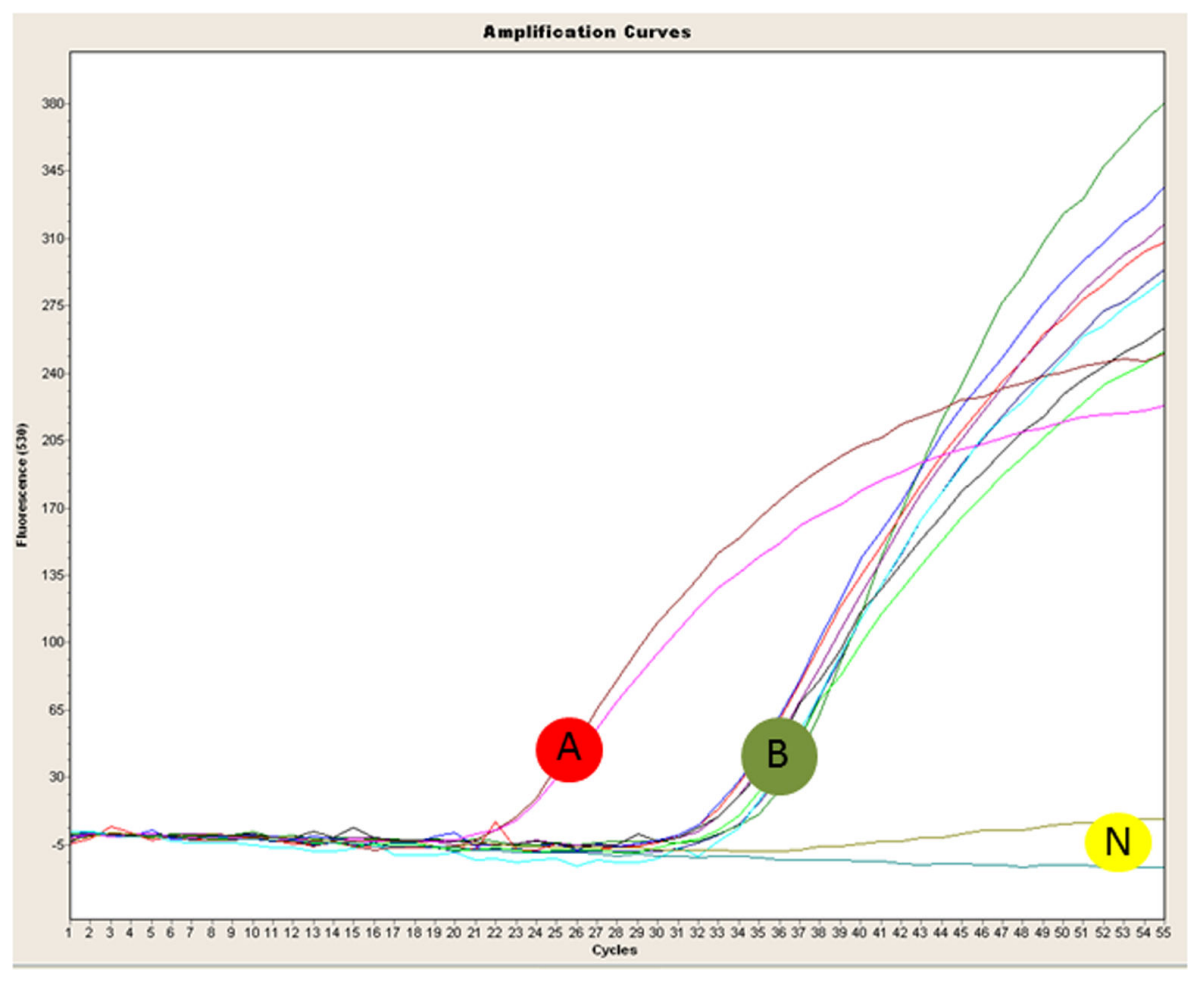
Graph from the Razor Ex BioDetection system after amplification of plasmid DNA (carrying the target gene sequence of corresponding primer set) and *Xylella fastidiosa* infected grape and oak plant samples (two samples for each plant), using the primer and probe set Xf.csp6. A: positive controls; estimated Ct value 24; B: *X. fastidiosa* infected grape (sample G1 and G2) and oak (sample O1 and O2) genomic DNA; estimated Ct values 33-35; N: non-template control (water). Each reaction was performed in two replicates.

Empty pouches filled with TaqMan qPCR reagents were used in place of the freeze-dried reagent pouches provided by the manufacturer. Only primer/probe set Xf.csp6 was used to detect *X. fastidiosa* from infected plant samples with the Razor Ex system. The Primer sets, Xf.CVC.fim1 and Xf.CVC.pib4 were not tested with samples infected with *Xfp* due to the categorization of *Xfp* as select agent in the USA at the time this research was conducted. However, all three primer and probe sets were tested using genomic *Xfp* DNA (isolate CVC50031) using the Razor Ex system and all were positive (Figure 4) with estimated Ct values of 24 (Xf.CVC.fim1), 24 (Xf.CVC.pib4) and 20 (Xf.csp6). All four infected plant samples (from grape and oak) were positive for *X. fastidiosa* using primer and probe set Xf.Csp6 (Figure 5) with estimated Ct values from 33-35. The reactions were performed in only one or two replicates due to the limited number of well slots (only 12) in the Razor Ex pouch. Razor Ex amplification and template DNA preparation results were reproducible. The entire protocol, from DNA extraction to final detection, takes approximately 30 min with no need for laboratory equipment.

## Discussion

*Xfp* CVC is a threat to the U.S. citrus industry. We developed and validated a field deployable, reliable and sensitive Razor Ex and qPCR assays for detection between *Xfp* and *X. fastidiosa*, using three primer and probe sets targeting the genes encoding fimbrillin, periplasmic iron-binding protein, and cobalamin. 

In biosecurity, quarantine and microbial forensics, assay specificity, accuracy and reliability are critical. The use of a multigene format maximizes reliability, specificity and broad range detection and minimizes the chances of false negative and positive results because each targeted gene-segment serves as an internal control for the other targeted gene-segments [25]. Two primer and probe sets specific for *Xfp*, selected after *in silico* evaluation, targeted genes encoding fimbrillin and a periplasmic iron-binding protein, and one set specific to all *X. fastidiosa* strains targeted the cobalamin synthesis gene. The three primer and probe sets were highly specific for their targets, and there was no cross reactivity with any other species in the exclusivity panels, which included important crops, vegetables, flowers, grasses, fruit trees and near neighbor microorganisms (Table 2). Primer and probe set Xf.csp6 detected twenty symptomatic *X. fastidiosa*-infected grape, oak, elm and mulberry plant samples collected from Texas and Oklahoma that did not carry the *Xfp* strain but were presumptively infected with *Xfp* closely related species, *X. fastidiosa* subsp. *multiplex* (causing agent of scorch disease in peach, almond and oaks) and *X. fastidiosa* subsp. *fastidiosa* (causing almond leaf scorch and Pierce’s disease of grapes) [3], while primer/probe sets Xf.CVC.fim1 and Xf.CVC.pib4 showed no reaction with these samples (Table 1). The primer-probe sets Xf.CVC.fim1 and Xf.CVC.pib4 detected the *Xfp* isolates as expected. *Xylella fatidiosa* Temecula was also not amplified using the Xf.CVC.fim1 and Xf.CVC.pib4 primer and probe set but amplified using primer and probe set Xf.csp6 (Table 1). 

Primer and probe sets Xf.CVC.fim1, Xf.CVC.pib4 and Xf.csp6 showed high sensitivity and efficiency, detecting as little as 1 fg of plasmid DNA and 1 fg (Xf.CVC.fim1) to 10 fg (Xf.CVC.pib4 and Xf.csp6) of *Xfp* genomic DNA. When genomic DNA from *Xfp* was mixed with extracts of orange rind and sharpshooter crude DNA, primer and probe set Xf.CVC.fim1 showed slight variation in their replicates, but only at ≤10 fg, indicating that orange rind and sharpshooter crude DNA cause little or no inhibition of qPCR sensitivity. Arif et al. [25], working with cotton leaf and soil extracts in PCR reactions containing genomic DNA of *Phymatotrichopsis omnivora*, also observed small differences among Ct values. However, they also indicated low reaction efficiency of 0.69 and 0.76 when primer set PoRPB2-2 was tested against *P. omnivora* genomic DNA spiked with cotton and soil extracts, respectively. 

Due to restrictions in the availability of *Xfp* infected plant samples, only the primer and probe set Xf.csp6, was tested with infected plant samples for pathogen detection using the Razor Ex. However, all three primer-probe sets were tested and validated with *Xfp* genomic DNA using the Razor Ex for on-field application. Compared to other on-site PCR instruments, the Razor Ex can be more easily transported because of its compact size and light weight (11 lb compared to the SmartCycler 74 lb) and was specifically designed for very rapid thermocycling. To perform the assays with these rapid cycling conditions and regular TaqMan reagents is not possible using traditional qPCR machines. The PCR cycling parameters can be loaded through barcodes (Figure 1) to operate the Razor EX means that the assay can also be used by other end users through direct scan from this publication. Commercially available Razor Ex pouches contain lyophilized PCR reagents to minimize contamination and circumvent cold storage. Because one aim of this research was to develop primers useful in different formats, we introduced commercially available TaqMan PCR components into empty pouches with disposable syringes. The entire assay required about 30 minutes, including approximately 10 minutes for sample preparation (DNA extraction) and 20 to 25 minutes for final detection. For further confirmation of the results for microbial forensics application, the biotinylated forward primer was used in Razor Ex to capture the amplified fragment using streptavidin magnetic beads, if required. Arif et al. [25] has demonstrated that biotinylated primer has no adverse effect on PCR amplification and sensitivity. The detection performances of the Razor Ex system and standard real-time qPCR technology (ABI 7300) were compared for specific detection of the causal agents of anthrax, brucellosis, tularemia, and plague [23] as well as influenza A viruses [24]. In our hands, the Razor Ex detected *P. omnivora* [25], *High plains virus* [31], *Xfp* and *Xf* with high assay specificity. The system generates reliable results and can be applied to phyto-sanitary diagnosis, in-field pathogen detection, and other applications in biosecurity and microbial forensics. Our results provide the framework for future development and validation of similar assays for other bacterial plant pathogens of high consequence. 
